# Supplementary figures and images for: Antiproliferative and Antimicrobial Potentials of a Lectin from Aplysia kurodai (Sea Hare) Eggs
Source: Mar Drugs. 2021 Jul 14;19(7):394. doi: 10.3390/md19070394 (PMC8306185; doi:10.3390/md19070394)

Supplemental figures

Suppl. Figure S1. Identification of N-terminal amino acid sequence of AKL-40

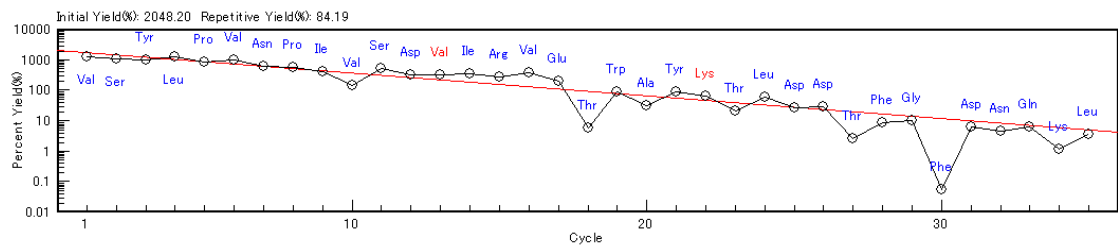

Supplement: Supplementary file 1 [file marinedrugs-19-00394-s001.zip › marinedrugs-1232738-supplementary.pdf]
